# Quantitative trait loci and candidate genes for yield-related traits of upland cotton revealed by genome-wide association analysis under drought conditions

**DOI:** 10.1186/s12864-023-09640-7

**Published:** 2023-09-07

**Authors:** Fenglei Sun, Yanlong Yang, Penglong Wang, Jun Ma, Xiongming Du

**Affiliations:** 1 State Key Laboratory of Cotton Biology, Institute of Cotton Research of the Chinese Academy of Agricultural Sciences, Anyang, 455000 China; 2 Hainan Yazhou Bay Seed Laboratory, Sanya, Hainan, 572000 China; 3 Research Institute of Economic Crops, Xinjiang Academy of Agricultural Sciences, Urumqi, 830091 China

**Keywords:** Upland cotton, Drought stress, Quantitative trait loci, Genome-wide association analysis, Candidate genes

## Abstract

**Background:**

Due to the influence of extreme weather, the environment in China’s main cotton-producing areas is prone to drought stress conditions, which affect the growth and development of cotton and lead to a decrease in cotton yield.

**Results:**

In this study, 188 upland cotton germplasm resources were phenotyped for data of 8 traits (including 3 major yield traits) under drought conditions in three environments for two consecutive years. Correlation analysis revealed significant positive correlations between the three yield traits. Genetic analysis showed that the estimated heritability of the seed cotton index (SC) under drought conditions was the highest (80.81%), followed by that of boll weight (BW) (80.64%) and the lint cotton index (LC) (70.49%) With genome-wide association study (GWAS) analysis, a total of 75 quantitative trait loci (QTLs) were identified, including two highly credible new QTL hotspots. Three candidate genes (*Gh_D09G064400*, *Gh_D10G261000* and *Gh_D10G254000*) located in the two new QTL hotspots, QTL51 and QTL55, were highly expressed in the early stage of fiber development and showed significant correlations with SC, LC and BW. The expression of three candidate genes in two extreme materials after drought stress was analyzed by qRT-PCR, and the expression of these two materials in fibers at 15, 20 and 25 DPA. The expression of these three candidate genes was significantly upregulated after drought stress and was significantly higher in drought-tolerant materials than in drought-sensitive materials. In addition, the expression levels of the three candidate genes were higher in the early stage of fiber development (15 DPA), and the expression levels in drought-tolerant germplasm were higher than those in drought-sensitive germplasm. These three candidate genes may play an important role in determining cotton yield under drought conditions.

**Conclusions:**

This study is helpful for understanding the regulatory genes affecting cotton yield under drought conditions and provides germplasm and candidate gene resources for breeding high-yield cotton varieties under these conditions.

**Supplementary Information:**

The online version contains supplementary material available at 10.1186/s12864-023-09640-7.

## Background

Cotton is the main natural fiber-producing crop and an important cash crop in China. Currently, there are four main cultivated cotton species, namely, A-genome diploid straw cotton (*Gossypium. herbaceum*: A1) and Asian cotton (*Gossypium. arboreum*: A2) and AD genome allotetraploid upland cotton (*Gossypium. hirsutum*: AD1) and island cotton (*Gossypium. barbadense*: AD2). Among them, upland cotton has a high yield and wide adaptability, and its output accounts for 95% of the total cotton production [1, 2]. Therefore, breeding upland cotton varieties with high yields is one of the goals of cotton breeding programs [3]. However, abiotic stress is one of the important factors affecting cotton production [3].

As an important cotton-producing region in China, Xinjiang is located in an arid and semiarid region in Northwest China, with little precipitation and agricultural water accounting for more than 90% of the total water in this region [4]. However, the growth and development of cotton are vulnerable to drought stress, and cotton yield and quality both decrease after drought stress [3, 5]. Cotton yield is closely related to fiber quality, and cotton fiber development is affected by drought stress, resulting in a yield loss of approximately 45% [6]. Water deficit at the flowering and boll stages can lead to decreased fiber strength, increased staple fiber content, and decreased quality, which then affect the yield of cotton [7]. Due to the frequent occurrence of extreme climate events, the impact of drought is increasingly severe. It is estimated that drought stress could reduce cotton production by 50–67% in 2050 [5, 8–10]. The yield characters of cotton mainly include boll number (BN), boll weight (BW), the seed cotton index (SC) and the lint index (LC), which are quantitative traits easily affected by the environment [3]. In previous studies, 4892 quantitative trait loci (QTLs) for important quantitative traits of cotton were reported, including more than 2226 QTLs for fiber quality and 991 QTLs for yield. These QTLs were associated with traits including BN, coat fraction, fiber length, fiber strength, macron value and the seed index [11, 12]. In conclusion, studies of QTLs in cotton, have mainly focused on fiber quality, while there have been fewer QTL studies focused on yield under conditions of drought stress. At present, most QTLs identified for drought tolerance traits are related to agronomic traits and physiological indicators, while most QTLs for yield have been identified under normal conditions. Saleem et al. used 524 simple sequence repeat (SSR) markers to conduct linkage analysis on F_2_ populations produced from drought-tolerant (B-557) and drought-sensitive (FH-1000) varieties, and a total of 22 QTLs related to drought resistance were detected [13]. Abdelraheem et al. treated backcross inbred lines (BIL) populations in the greenhouse under PEG-induced drought stress, and detected two QTLs each for related to plant height, stem fresh weight and root weight using SSR markers [14]. Additionally, 165 drought-tolerant sites in a recombinant inbred line (RIL) of upland cotton were identified using resistance gene analog-amplified fragment length polymorphism (RGA-AFLP) and genotyping by multiplex sequencing-single nucleotide polymorphism (GMS-SNP) markers under greenhouse and field conditions [15]. Using 403 SSR markers, 15 stable abiotic stress QTLs were located in a RIL population of island cotton [10]. With the development of sequencing technology, cotton genomes have been sequenced and published. Some QTLs related to yield traits have been discovered through genome-wide association analysis using the reference cotton genome [16–19]. These QTLs were identified under normal environmental conditions using the single variant association genome-wide association study (GWAS) approach, so there are still some stable loci to be analyzed.

In this study, 188 upland cotton germplasms were planted in multiple environments for many years and subjected to drought stress treatment. Phenotypic analysis of three main yield traits and five agronomic traits closely related to yield traits was performed. Stable QTLs related to yield composition were identified after drought stress in multiple environments using the multisite random-SNP-effect mixed linear model. Combined with transcriptomic data analysis, the expression patterns of selected candidate genes were studied, and the key genes affecting the yield of cotton after drought stress were predicted. The results of this study are helpful for better understanding the genetic structure of yield traits after drought stress and provide molecular markers and candidate genes for breeding drought-tolerant and high-yield cotton varieties.

## Result

### Analysis of phenotypic variation in 8 traits

**Fig. 1:**
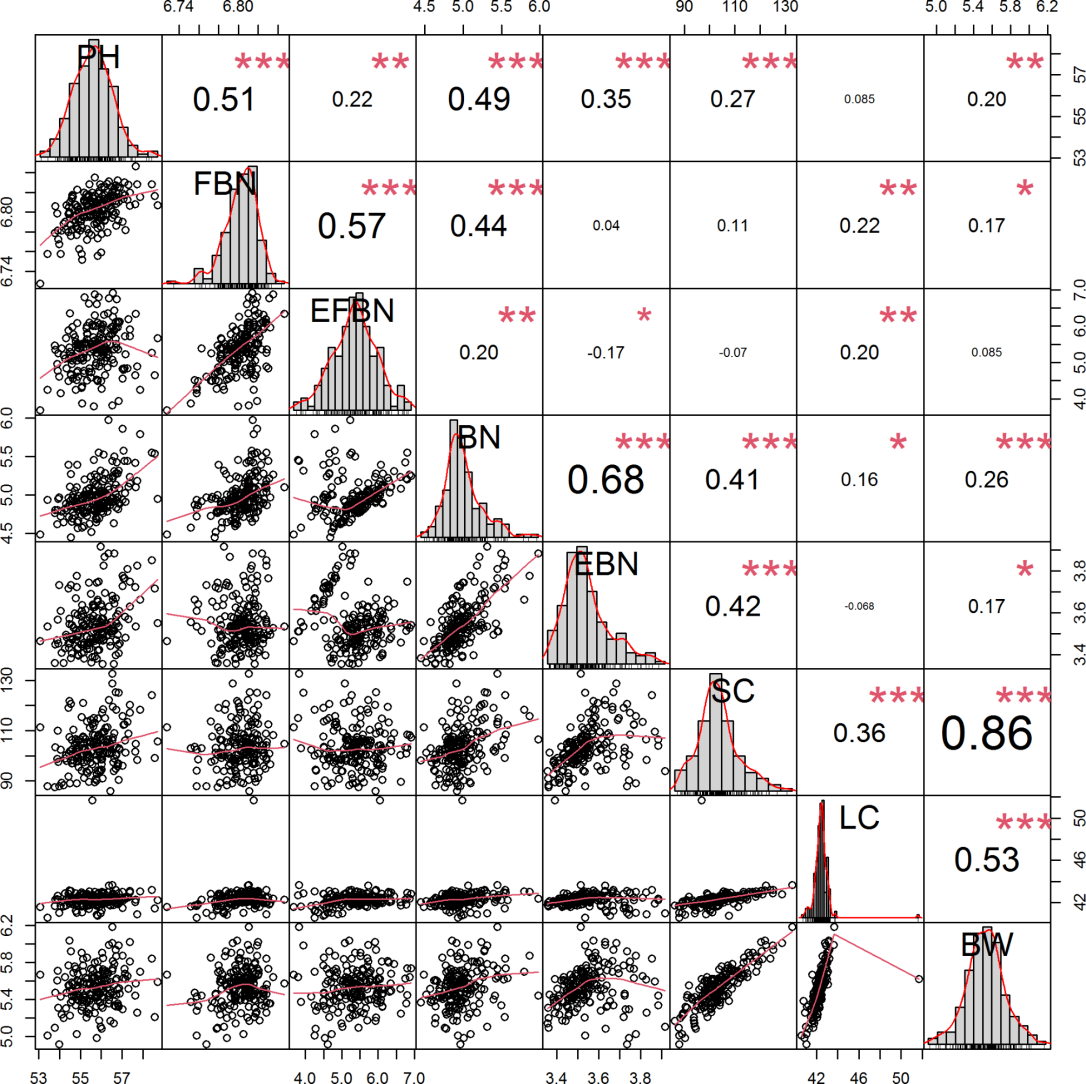
Correlation analysis of the 8 characters. Significant differences: *** (p < 0.001), ** (p < 0.01), and * (p < 0.05). PH: plant height, FBN: fruit branch number, EFBN: effective fruit branch number, BN: boll number, EBN: effective boll number, SC: seed cotton, BW: single-boll weight, LC: lint cotton

The phenotypic variation in 8 traits in 188 upland cotton germplasms was analyzed under two conditions in three environments. The traits analyzed included five agronomic traits, such as plant height (PH) and fruit branch number (FBN), and three yield-related phenotypic traits including SC, LC and BW (Table S4). The results showed that there was a great difference in population materials after drought stress treatment. Under the two treatment conditions, the PH of the five agronomic traits was 28-103.2 cm, FBN was 3-25.4, effective fruit branch number (EFBN) was 0.6–19, BN was 1-17.8, and EBN was 0.6–22.8. The three yield traits SC, LC and BW were 48.93-223.83 g, 4.56–83.13 g and 2.74–11.19 g, respectively (Table S4). The coefficients of variation (CVs) for BN and EBN were the greatest, at 2.29–57.09% and 2.92–57.07%, respectively. All 8 traits were normally distributed (Figure S1). The distribution of maximum and minimum values of the eight traits under normal conditions was greater than that under drought stress (Table S4). The results of variance analysis under the two conditions revealed significant differences between the eight traits after water treatment (Table S3). Correlation analysis revealed highly significant correlations between the 8 traits, and BW, SC and LC were extremely significantly positively correlated (Fig. 1). The best linear unbiased estimation (BLUP) results in multiple environments showed that the BLUPs of the eight phenotypic values were 53.06–67.7 cm, 6.73–10.41, 3.68–8.62, 4.45–9.87, 3.36–7.3, 98.08-134.55 g, 40.57–51.7 g and 4.91–6.73 g, respectively (Table S4). The effects of genotype (G), environment (E) and their interaction on the eight traits under the two water treatment conditions were assessed using analysis of variance (Table S3). The results showed that the eight traits were influenced by the interaction between genotype and environment. The generalized heritability (H^2^) of the 8 characters (Table S3) was estimated based on the phenotypic traits in 3 environments. Under drought stress, the heritability of EFBN was the highest (89.47%), followed by those of SC and BW (80.81% and 80.64%, respectively), and that of PH (67.31%) was the lowest (Table S3).

### Location analysis of the 8 characters examined under drought stress

**Fig. 2:**
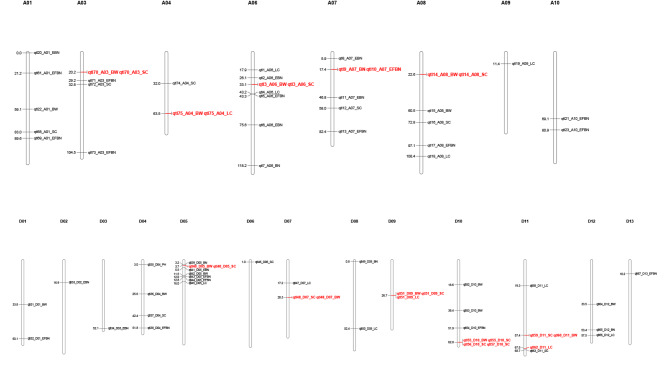
Distribution of candidate QTLs for the 8 examined traits. The left side of the chromosome shows the physical distance of the QTL. QTL hotspots with high confidence are marked in red

Eight phenotypic traits and BLUP phenotypic values were analyzed under drought stress by using a multisite random-SNP-effect mixed linear model based on the genotype data of 412,856 high-quality SNPs. A total of 1329 quantitative trait nucleotides (QTNs) significantly correlated with the 8 examined traits were identified on 26 chromosomes. Using 500 kb as the LD threshold, we combined overlapping QTLs and BLUP values and finally identified 1228 QTLs for the 8 traits in 3 environments (Table S5). To avoid false positives arising from QTLs with few associations, QTLs identified in at least two environments were considered to be stable QTLs, so a total of 75 stable QTLs were identified. Among them, 1 was from PH, 17 from EFBN, 9 from BN, 10 from EBN, 19 from SC, 12 from LC and 17 from BW (Fig. 2 and Table S5).

Among the 75 QTLs identified, 11 QTLs were simultaneously identified as associated with two or more traits (Table S6), such as LC and BW, and two QTLs significantly positively correlated with two traits (R = 0.86) were detected. The identified QTLs were widely distributed on 26 chromosomes, and there were more QTLs in the At subgenome than in the Dt subgenome (Table S6, Figure S2). Three homologous QTLs were identified between the A and D subgenomes (Table S7).

QTLs detected for two or more traits and in more than two environments were considered QTLs with high confidence, and a total of 11 QTLs with high confidence were identified (Fig. 2, Table S6). QTL51 was associated with three yield traits (SC, LC and BW) in three environments, and QTL55 was associated with BW and SC in three environments. In addition, seven QTLs were associated with two traits, and QTL60 was associated with SC and LC in the two environments, suggesting that these QTLs play an important role in the yield of upland cotton under conditions of drought stress.

### Identification of candidate genes in QTL regions

Information about the genes within candidate QTL regions was extracted from the published upland cotton reference genome (TM-1). A total of 2945 genes were identified across all candidate QTLs, including 69 PH-related genes, 528 EFBN-related genes, 481 BN-related genes, 661 EBN-related genes, 587 SC-related genes, 536 LC-related genes and 518 BW-related genes. After the genes with low expression were filtered out, a total of 2736 candidate genes were identified, of which 51 were related to PH, 406 to EFBN, 393 to BN, 532 to EBN, 470 to SC, 448 to LC, and 439 to BW (Table S8). Gene Ontology (GO) analysis was performed on all screened candidate genes for each trait to identify their relevant functions (Figure S4). The results showed that candidate genes were enriched in different biological processes in different QTL regions associated with different traits. For example, genes associated with SC were mainly enriched in the processes of carbon and lipid metabolism, such as lipase activity and carrot-like dioxygenase activity. Genes associated with LC were mainly enriched in proline metabolism and starch and sucrose metabolism and in processes related to fiber development, such as actin filament tissue and calmodulin binding biological processes. Genes associated with BW were mainly enriched in photosynthesis, plant hormone signal transduction, carbon metabolism, glycolysis/gluconeogenesis and other biological processes, as well as in actin-dependent ATPase activity related to fiber development. Under drought conditions, the biological functions enriched in the identified QTL regions were related to the development process of yield components.

### Key QTL and gene mining of yield-related traits under drought stress

**Fig. 3:**
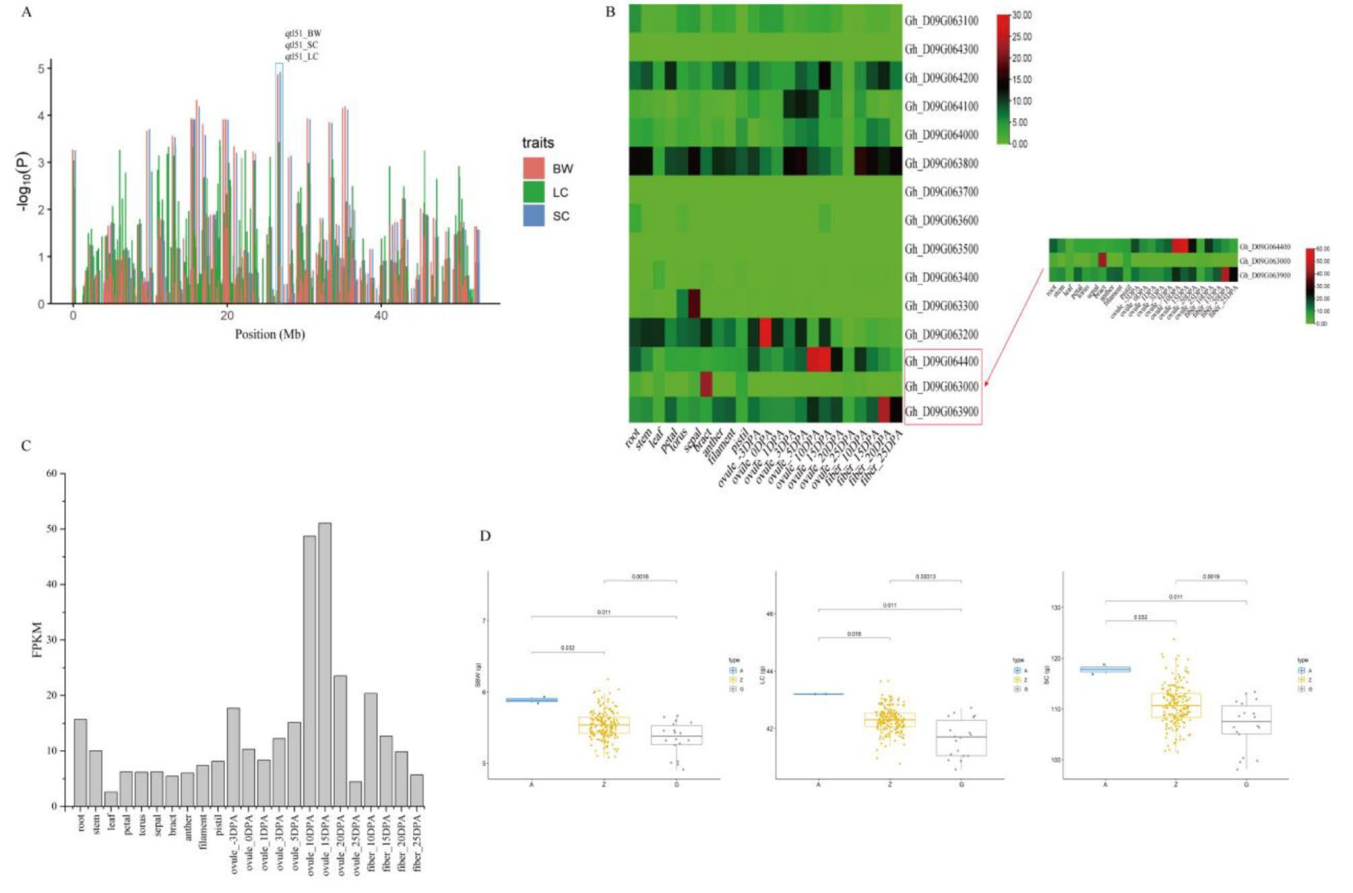
QTL hotspots and candidate genes on chromosome D09. **(a)** QTL hotspots associated with BW, SC and LC on chromosome D09. **(b)** Heatmaps of candidate gene expression for three QTLs. **(c) ***Gh_D09G064400* expression analysis in different tissues. **(d)** Differential analysis of recent QTN phenotypic values for this gene

Among the 11 QTLs identified with high confidence, QTL51 and QTL55 were associated with three and two yield traits, respectively (Fig. 2). Through analysis of transcriptome data obtained from different tissues, most of the 15 genes in the QTL51 region were found to be expressed in fiber tissues or ovules during fiber development (Fig. 3b), and a gene encoding protein phosphatase 2 C (*Gh_D09G064400*) was identified in this region. *Gh_D09G064400* is homologous to Arabidopsis protein phosphatase 2 C and is highly expressed in fibers and ovules, especially in the early stages of fiber development (Fig. 3c). Moreover, the expression of *Gh_D09G064400* was significantly higher at 12 and 24 h after drought stress (Figure S3). The QTN (GX370348), which is most closely related to the protein phosphatase 2 C protein gene, was selected to assess the correlation between this gene and phenotypic traits affecting yield. Comparison of the SC phenotypes of the three genotypes at this site revealed that the SC value of genotype A was significantly higher than that of both genotype G and the heterozygous genotype and that the SC value of genotype G was the lowest. Moreover, comparison of the LC and BW phenotypes of the three genotypes revealed results consistent with those observed for SC (Fig. 3d). Furthermore, transcriptome data showed that this gene was highly expressed in developing fiber and ovules, indicating its potential role in enhancing SC, LC and BW. Another stable locus, QTL55, was associated with BW and SC etc. yield traits. A total of 87 genes were identified in this QTL region. Transcriptome data analysis revealed that some of these genes were highly expressed in fiber tissues or ovules during fiber development (Figure S5). Two candidate genes were screened: *Gh_D10G254000*, which is a member of the actin gene family and homologous to the actin gene of Arabidopsis thaliana, and *Gh_D10G261000*, which is a protein phosphatase 2 C protein gene. Both of these genes are highly expressed in ovules at the early stage of fiber development (Figure S5c). Similarly, the most recently identified QTN (GX69396) was selected for analysis, and it was found that the SC and BW values of the G genotype were significantly higher than those of the A genotype (Figure S5d), indicating that these two candidate genes also play an important role in yield-related phenotypes.

**Fig. 4:**
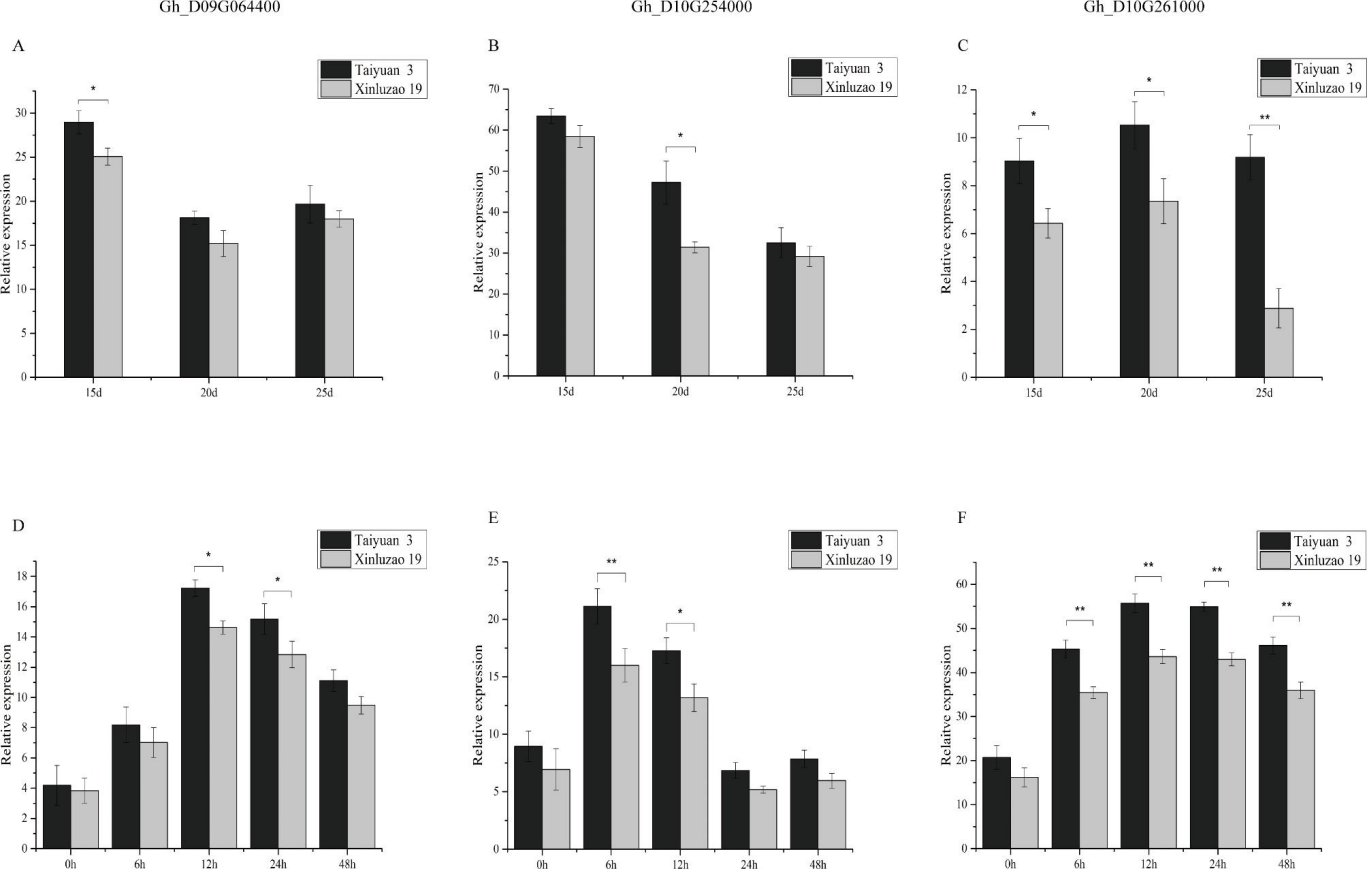
Three candidate genes were analyzed by qRT-PCR in two extreme materials. **A**, **B**, and **C** show expression at 15, 20, and 25 DPA of fiber development, respectively, while **D**, **E**, and **F** show expression after drought stress

The extreme drought-tolerant varieties Taiyuan 3 and Xinluzao 19 were subjected to drought stress, and the expression levels of the three candidate genes were analyzed. The results show that the three candidate genes identified in the drought-tolerant Taiyuan 3 variety were highly expressed following drought stress, and the expression of these genes was significantly higher in varieties with high drought-stress tolerance than in those with low drought-stress tolerance (Fig. 4). At 15, 20 and 25 DPA of fiber development, the expression levels of the three candidate genes in the materials with high drought-stress tolerance were also significantly higher than those in the materials with low drought-stress tolerance (Fig. 4), and expression levels were high during the early stage of fiber development.

## Discussion

The impact of drought stress on crops is ultimately reflected in yield, and increasing crop yield is currently an important goal of crop breeding [3]. Cotton is an important cash crop in China, and its yield components mainly include BN, BW, BW and LC [3]. These traits are determined by the interaction between diverse genetic loci and the environment [20]. The degree of genetic diversity identified in previous studies was similar to that found in this study [21, 22]. Therefore, to reduce the impact of the environment on the results of GWASs, phenotypic data were collected from three environments in two years and combined to estimate BLUP values in order to improve the reliability of the data. The results of statistical analysis of the phenotypic data from different treatments in different environments showed that the observed variation in the eight traits ranged from 0.26 to 57.09% (Table S4). Another important influencing factor in association analysis is heritability [22]. The degree of heritability of genetic traits is usually defined as H^2^, and when H^2^ is greater than 50%, the heritability is considered high [23]. Drought stress, as an environmental factor, affecteds the H^2^ of the studied traits. BN has a significant influence on the yield of cotton per plant and previous studies reported that BN is an important factor affecting the yield of cotton fiber [3, 24]. In this study, statistical analysis of phenotypic traits showed that BN had a lower H^2^ value (68.37%) and a higher CV (Table S4) after drought stress, indicating that the trait was greatly influenced by the environment. However, the SC of cotton affected by BW and LC, so SC can be used as an indicator of two important characteristics of cotton yield increase. The H^2^ of BW and LC in this study was 80.64% and 70.49%, respectively (Table S3), both of which are high H^2^ levels but are lower than those previously reported [16, 25]. However, analysis of variance showed that genotype, environment and their interaction led to significant differences in the interpretation of these traits (Table S3). At present, most studies on yield have focused on the improvement of LC [16, 25], but there have been relatively few studies focused on SC and BW. Fibers are grown on ovules and play a key role in the process of fiber development. Therefore, phenotypic data analysis showed that increasing SC and BW values may improve fiber production. A correlation between cotton fiber yield and cotton seed size also appeared during the evolution process [26, 27]. In this study, the phenotypic analysis results revealed significant positive correlations between SC, LC and BW (Fig. 1), indicating that under drought conditions, high BW and SC were conducive to improving cotton fiber yield, so as to search for candidate genes to improve cotton yield.

Advances in sequencing technology and the open access of whole-genome data have facilitated the identification of many QTLs and genes related to cotton yield [16–19]. However, Xinjiang, as the main producing area of cotton, is susceptible to drought stress, and because the yield is a complex quantitative character, it is easily affected by the environment. Therefore, we used a mixed linear model of multi-site random-SNP-effect for analysis, and previous studies also indicated that this model could improve the stability and efficacy of association analysis [28]. In this study, a total of 188 upland cotton germplasms were treated with drought stress in multiple field environments. With a GWAS using the multisite random SNP mixed linear model, stable QTLs and candidate genes associated with 8 traits were analyzed after drought stress.

In this study, a total of 1229 QTNs were identified under conditions of drought stress; however, the effect of some QTNs was small (R^2^ ≤ 0) (Table S5), which may be indicative of a false positive. Furthermore, some QTNs were detected only in one environment, and their R^2^ values were low (Table S5). Therefore, we defined QTLs that were detected simultaneously in more than two environments after drought stress as candidate QTLs to ensure their authenticity. A total of 75 QTLs were identified (Table S6), among which two QTLs had been reported previously under normal conditions. In this study, two new QTL hotspots were identified (Table S6), and these two new QTLs were associated with three yield traits (LC, SC, and BW), providing new target loci for the genetic improvement of cotton yield traits under drought conditions.

In this study, a total of 102 genes were identified in two candidate QTL regions (Fig. 3 and Figure S5). Transcription data expression analysis results showed that most of these genes were highly expressed in the early stage of fiber development, and three candidate genes were screened out (Fig. 3c and Figure S5c). Analysis of the QTN near these three candidate genes revealed three distinct categories, among which there were significant differences in phenotypes (Fig. 3d and Figure S5d). In extremely drought-tolerant germplasm, the expression of three genes was induced following drought stress, and it was higher in germplasm with high drought-stress tolerance than in germplasm with low drought-stress tolerance (Fig. 4). Moreover, at 15, 20 and 25 DPA of fiber development, the expression levels of these genes were higher in germplasm with high drought tolerance (Taiyuan 3) than in germplasm with low drought tolerance (Xinluzao 19) (Fig. 4). Studies on these three genes found that *Gh_D10G261000* and *Gh_D09G064400* encoded protein phosphatase 2 C, which may be involved in brassinosteroid-mediated related pathways [29]. Additionally, *Gh_D10G254000*, a member of the actin gene family, is homologous to Arabidopsis actin genes [30], may be involved in the mitogen-activated protein kinase (MAPPK) and calcium transduction signaling pathways. Therefore, these three candidate genes may have a positive effect on improving cotton yield under drought conditions. The specific role of these three candidate genes in fiber development still needs to be studied.

## Conclusions

It is important to identify loci and candidate genes related to yield traits under drought conditions in order to improve cotton yield in arid areas. In this study, data related to eight phenotypic traits were collected from 188 cotton germplasms under drought stress in three environments. Genetic analysis showed that the three yield-related traits had high heritability and were significantly positively correlated. GWAS analysis of samples subjected to drought stress revealed 75 stable QTLs, including 11 high-confidence QTLs and two new QTL hotspots. A total of 102 genes were identified in these two QTL regions, and three candidate genes were finally screened by RNA-seq and qRT-PCR analysis. *Gh_D10G261000* and *Gh_D09G064400* were located in the same QTL region (QTL55) and were highly expressed under conditions of drought stress and at 20 days after fiber development. *Gh_D10G254000*, located in QTL51, is an actin-encoding gene that is also highly expressed under drought stress and is highly expressed at the 15 DPA of fiber development. These results provide excellent sites and candidate gene resources for cultivating high-yield cotton germplasm under drought conditions.

## Materials and methods

### Plant material

In 2020 and 2021, 188 upland cotton germplasms (Table S1) were planted in three natural environments in Korla and Shihezi, Xinjiang (Shihezi and Korla in 2020, Korla in 2021) [31]. The planting management followed the local field management. All germplasms were planted under a completely randomized block (RCBD) experimental design, with two treatments (normal control and drought stress) in each environment and two replicates of each treatment. Each germplasm was planted in a 2-m-long plot with a row spacing of 66 + 10 cm (wide and narrow configuration) and 10-cm plant spacing, and each germplasm was planted in two rows. Normal irrigation during the whole growth period was considered the normal treatment, and in the drought stress treatment, irrigation was artificially stopped twice in the flowering and boll stages to impose drought stress.

### Phenotypic data collection and data analysis

Three yield traits (BW, SC and LC) and five agronomic traits (PH, FBN, EFBN, BN and EBN) were measured in cotton plants with uniform growth after mature boll opening in late September. For each germplasm, 20 bolls were collected from the middle of two rows of cotton plants for weight measurement, and three indexes, single boll weight, seed cotton weight and lint weight, were obtained. The main investigation methods used were based on the Specification and Data Standard of Cotton Germplasm Resources Description [32].

In this study, phenotypic analysis of 8 indicators was performed for the two treatments in two environments. Descriptive statistical analysis was conducted for the 8 indexes of the population germplasm using the description function in the Hmisc package of R, and the degree of dispersion of all materials was analyzed by the CV of the observed data when comparing different traits and attributes. Population variance analysis was performed in R to evaluate genotype (G), environment (E), and gene-environment interaction (G×E) effects. Generalized heritability was calculated with the following formula: H^2^ = V*g*/(V*g* + V*ge*/n + V*r*/n), where V*g* represents the genotype variance, V*ge* represents the variance of genotype and environment, V*r* represents the residual, and n represents the replicate [33]. If the result of the F test was significant, a least significant difference (LSD) test for multiple comparisons was used (P < 0.05 was defined as significant) [34, 35]. The density distribution of phenotypic values was mapped by R software.

### Genotyping and SNP calling

In a previous study, DNA was extracted from tissue samples of 188 young leaves of cotton resource materials for resequencing [36]. The original sequence reads obtained by sequencing were filtered, and the adaptor sequences and low-quality reads with more than 10% N bases were removed to obtain clean reads. Clean double-terminal resequencing reads were mapped to the TM-1 (*G. hirsutum*) reference genome using Burros-Wheeler alignment (BWA) software [37]. After Picard’s Mark duplicate tool was used to remove duplicate reads, GATK software was used to identify SNPs and indels [38]. SNPs with retention integrity > 80% and a minor allele frequency (MAF) < 0.05 were filtered from the original SNP data set, and a dataset containing 412,856 high-quality SNPs was finally obtained for subsequent QTL screening.

### Genome-wide association analysis and QTL identification

The multisite random-SNP-effect mixed linear model in “mrMLM” was used to identify QTLs for each trait (including traits measured under drought stress and normal conditions, as well as calculated BLUP values) [39]. In mrMLM analysis, the genetic relation (K) matrix was obtained directly, and the Q + K statistical model was selected in GWAS analysis. We used the 500-kb regions upstream and downstream of significant SNP sites as QTLs and combined overlapping QTLs into a single QTL to determine the number of QTLs. In this study, QTLs identified in multiple environments were considered stable QTLs. QTLs were named using the following formula: q + trait abbreviation + chromosome number + QTL number.

### Identification and qRT-PCR analysis of candidate genes

To identify potential candidate genes, gene annotation information in the QTL interval was extracted from the annotated genes in the upland cotton TM-1 reference genome (CRI v1) [40], and the gene sequence was analyzed by BLAST. Candidate genes in relevant regions were compared with genes in different genome databases (GO and KEGG) [41] to screen genes. Then, the candidate gene sequences within the QTL were analyzed to determine whether there was a mutation site and, if present, to analyze whether the mutation led to differences in the expression of related genes. To identify which candidate genes were associated with yield composition, transcriptomic data were used to analyze the fibrous tissue expression levels of roots, stems, leaf petals, and ovules (10, 20 and 25 DPA ovules) and post flowering − 3, 0, 1, 3, 5, 10, 15, 20 and 25 DPA (PRJNA490626 [42]). Among the experimental materials, Taiyuan 3, a variety with high yield and relatively high drought tolerance, and Xinluzao 19, a variety with low yield and drought sensitivity, were selected. Leaf tissues were collected for RNA extraction after 0, 6, 12, 24 and 48 h of drought stress. Additionally, fiber tissues were collected 15, 20 and 25 DPA after fiber development for RNA extraction, which was repeated three times. RNA was extracted from the collected samples using a total RNA extraction kit (Tiangen, China). cDNA was synthesized by using a One-Step RT-PCR Kit. Based on the cDNA sequence of candidate genes, specific primers were designed for real-time quantitative fluorescent PCR detection (qRT-PCR) analysis (Table S2). Real-time PCR amplification was performed on the ABI 7500 Fast system with three replicates for each sample collected. *GhUBQ7* was used as the internal reference gene, and the results were quantitatively analyzed by the 2^−ΔΔCt^ method [43].

### Electronic supplementary material

Below is the link to the electronic supplementary material.

Supplementary Material 1

Supplementary Material 2

## Data Availability

This published article and its supplementary information files include all data generated or analyzed during this study. Resequencing data in this study were stored in NCBI SRA under PRJNA605345 [35]. In this study, mRNA-seq data were stored in NCBI SRA under PRJNA490626 [41].
