# Supplementary material for: Quantitative trait loci and candidate genes for yield-related traits of upland cotton revealed by genome-wide association analysis under drought conditions
Source: BMC Genomics. 2023 Sep 7;24:531. doi: 10.1186/s12864-023-09640-7 (PMC10485960; doi:10.1186/s12864-023-09640-7)
Supplement: Supplementary file 2 — Supplementary Material 2 [file 12864_2023_9640_MOESM2_ESM.docx]

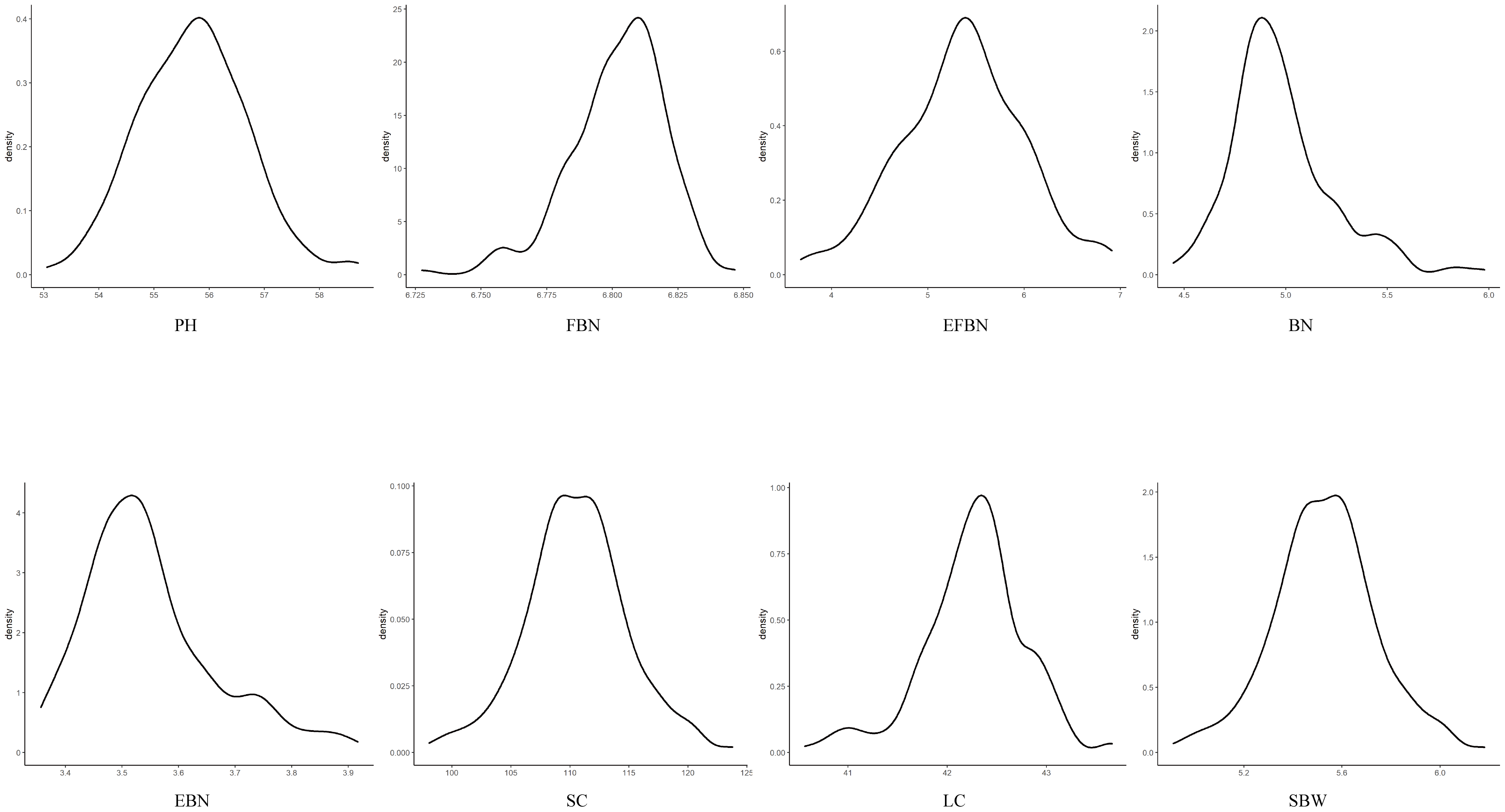


Figure S1 Density distribution of 8 traits.


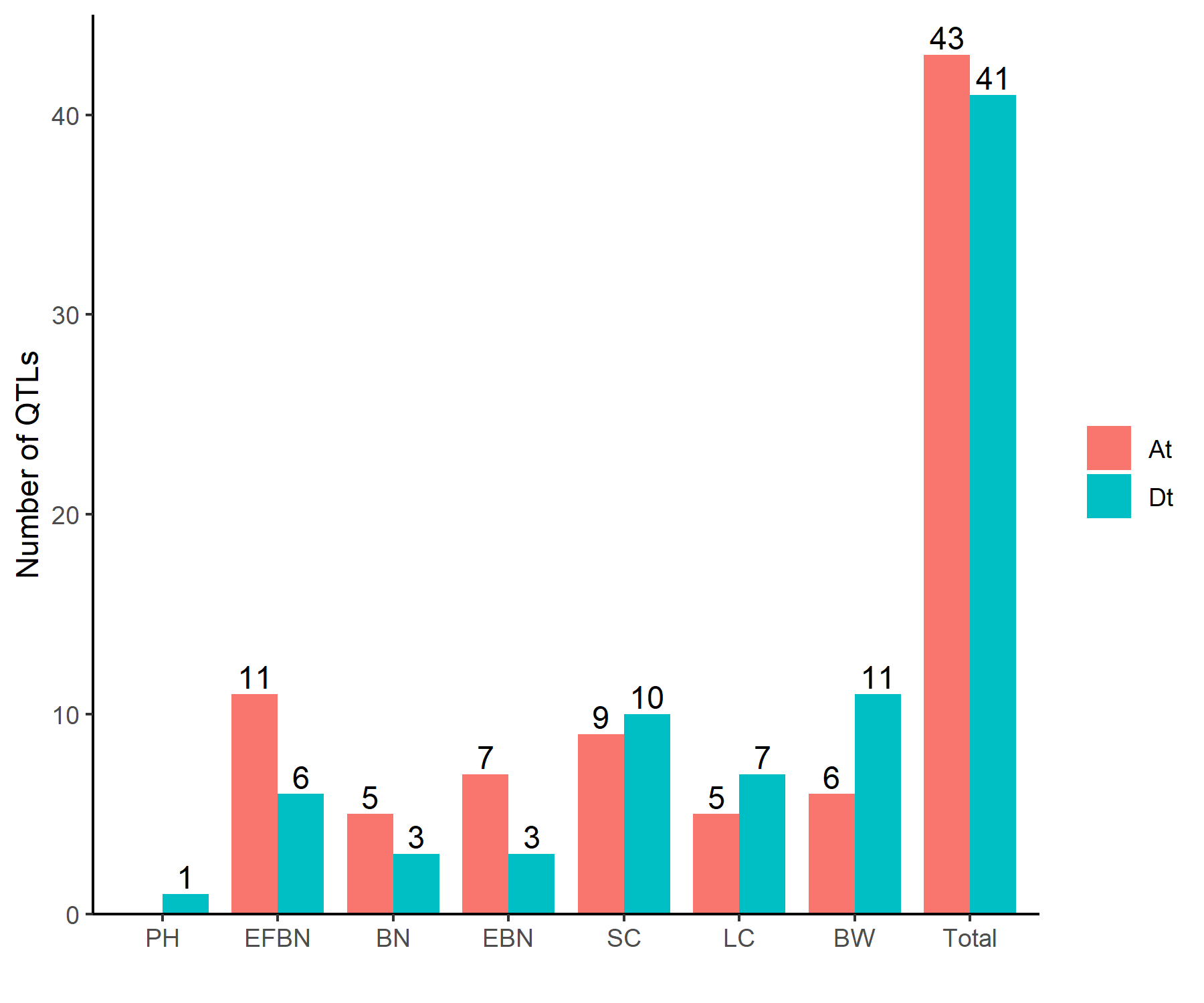


Figure S2 The number of candidate QTLS associated with 8 traits on the At and Dt subgenome.


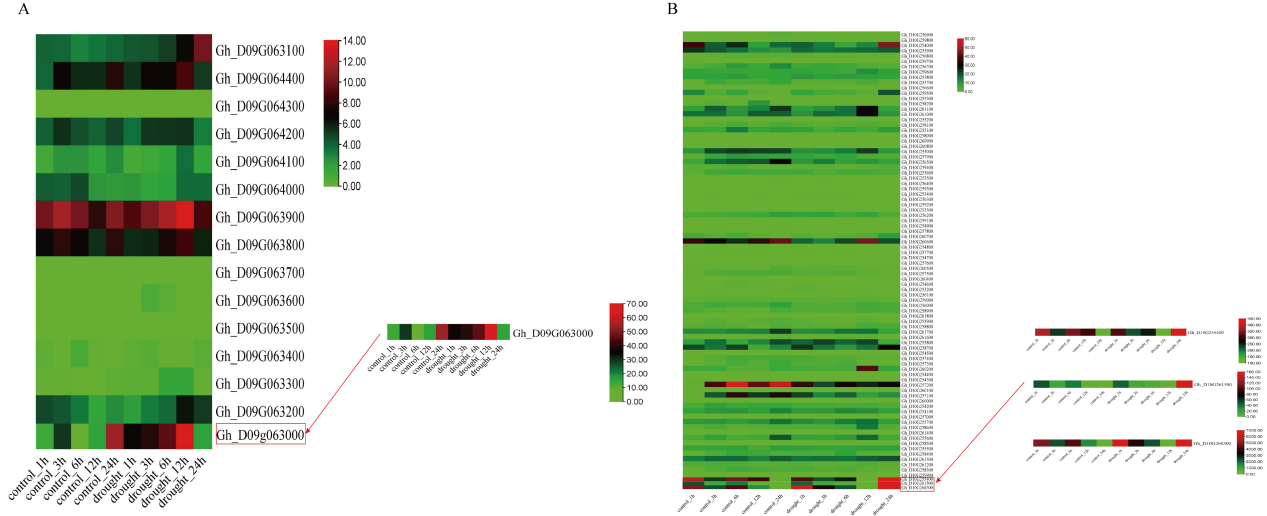


Figure S3 Heat map analysis of all genes in two candidate QTL hot spots (QTL51 and QTL70) after drought stress.


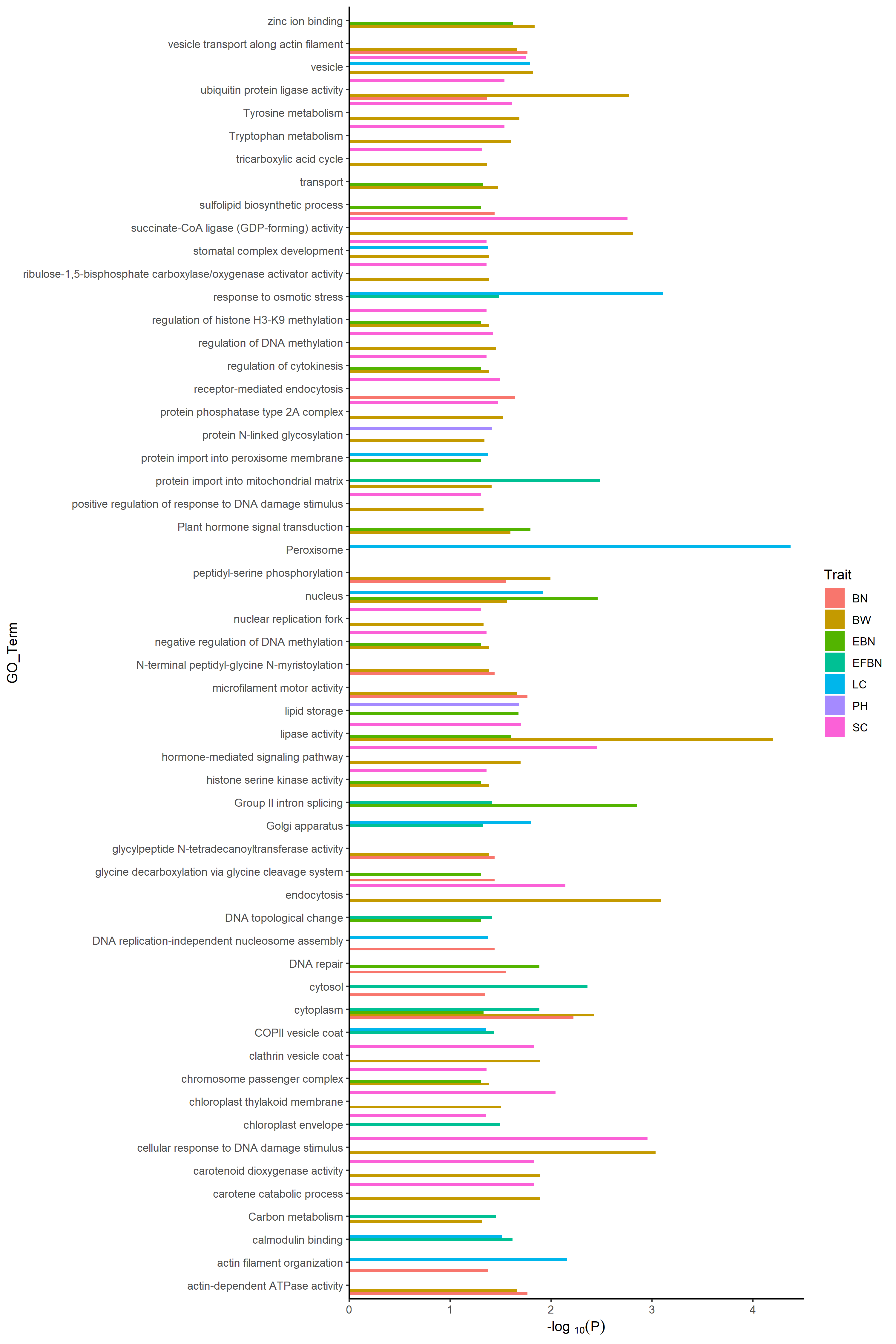


Figure S4 GO analysis of 8 traits candidate genes.


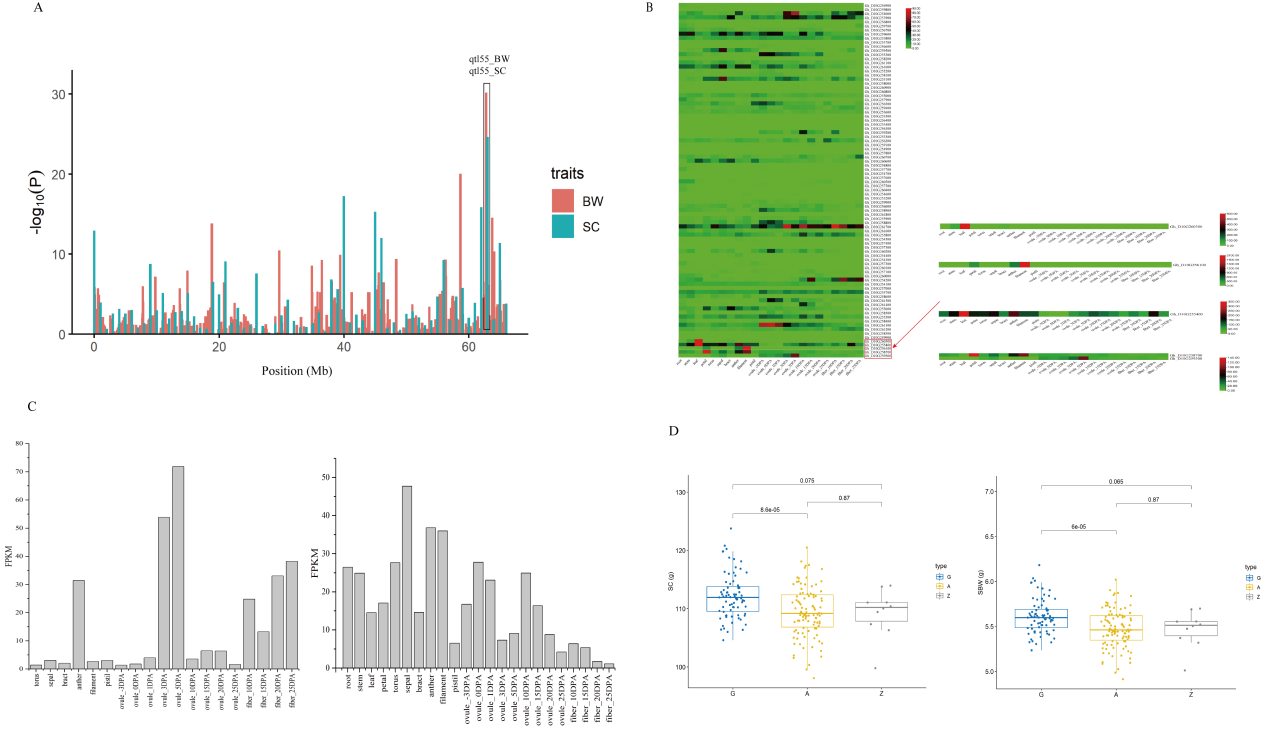


Figure S5 QTL hotspots and candidate genes on chromosome D10. a. QTL hotspots associated with BW and SC on chromosome D10. b. Heat maps of candidate gene expression for three QTL. c. Expression analysis of Gh_D10G254000 and Gh_D10G261000 gene in different tissues. d. Analysis of recent QTN phenotypic values difference between these two genes.
